# Implementation of a guideline for local health policy making by regional health services: exploring determinants of use by a web survey

**DOI:** 10.1186/s12913-017-2499-2

**Published:** 2017-08-15

**Authors:** Theo J.M. Kuunders, Monique A.M. Jacobs, Ien A.M. van de Goor, Marja J.H. van Bon-Martens, Hans A.M. van Oers, Theo G.W.M. Paulussen

**Affiliations:** 1 Tilburg University, Tilburg School of Social and Behavioral Sciences, Tranzo Scientific Center for Care and Welfare, PO Box 90153, 5000 LE Tilburg, The Netherlands; 2 Regional Health Service, ‘GGD Hart voor Brabant’, ‘s-Hertogenbosch, PO Box 3024, 5003DA Tilburg, The Netherlands; 3 TNO, Innovation for Life, Organization for Applied Scientific Research, PO Box 2215, 2301CE Leiden, The Netherlands; 4 Trimbos Institute, Netherlands Institute of Mental Health and Addiction, PO Box 725, 3500AS Utrecht, The Netherlands; 5 National Institute for Public Health and the Environment, PO Box 1, 3720BA Bilthoven, The Netherlands

**Keywords:** Local health policy, Regional health service, Guideline use, Web based survey, Implementation determinants, Implementation strategy, The Netherlands

## Abstract

**Background:**

Previous evaluation showed insufficient use of a national guideline for integrated local health policy by Regional Health Services (RHS) in the Netherlands. The guideline focuses on five health topics and includes five checklists to support integrated municipal health policies. This study explores the determinants of guideline use by regional Dutch health professionals.

**Methods:**

A web survey was send to 304 RHS health professionals. The questionnaire was based on a theory- and research-based framework of determinants of public health innovations. Main outcomes were guideline use and completeness of use, defined as the number of health topics and checklists used. Associations between determinants and (completeness of) guideline use were explored by multivariate regression models.

**Results:**

The survey was started by 120 professionals (39%). Finally, results from 73 respondents (24%) were eligible for analyses. All 28 Dutch RHS organizations were represented in the final dataset. About half of the respondents (48%) used the guideline. The average score for completeness of use (potential range 1–10) was 2.37 (sd = 1.78; range 1–7). Knowledge, perceived task responsibility and usability were significantly related to guideline use in univariate analyses. Only usability remained significant in the multivariate model on guideline use. Only self-efficacy accounted for significant proportions of variance in completeness of use.

**Conclusions:**

The results imply that strategies to improve guideline use by RHSs should primarily target perceived usability. Self-efficacy appeared the primary target for improving completeness of guideline use. Methods for targeting these determinants in RHSs are discussed.

**Electronic supplementary material:**

The online version of this article (doi:10.1186/s12913-017-2499-2) contains supplementary material, which is available to authorized users.

## Background

The development and implementation of public health policies in the Netherlands is largely delegated to local authorities. In this process, municipalities are supported by Regional Health Services (RHSs). RHSs focus on three main prevention areas of Infectious Disease, Youth Health, and lifestyle related Health Promotion. Typical RHSs professions are doctors, nurses, health promoters, health scientists, policy officers, and epidemiologists. Their work consists of directly client-oriented activities (e.g. information on sexually transmitted diseases; intervention for obesity prevention, health education in schools), advisory for policy development and of collecting statistical information (monitoring of regional - and local trends in health and disease) to provide input for regional - and local policy advice. RHSs can either have a regional scope and serve multiple municipalities or serve a single (urban) municipality.

Since 2006, the Dutch Ministry of Health has equipped municipalities and RHSs with national guidelines for the planning and implementation of their public health policies [1]. Four different guidelines, incorporating recommended interventions to address smoking [2], obesity [3], alcohol abuse [4], and depression [5] were issued separately and were published sequentially within a period of two years. Preliminary evaluation indicated unsatisfactory results concerning the guidelines’ implementation and led to a revised, more extended and comprehensive Guideline for Local Health Policy (hereafter ‘guideline') [6]. The new guideline integrated the four separate guidelines, and added the topic ‘sexual health’, and new tools (checklists) for developing cross-sectoral public health policies. The overall purpose of the guideline is to stimulate the use of evidence in this planning process [7]. The guidelines’ health topics and checklists serve different, though related purposes. The health topics are about selection and application of exemplary interventions, while the checklists provide leads for improving collaboration and commitment among those participating in the planning of local health policies. RHS policy officers are called upon to use the checklists containing leads for health policy planning, and evaluation. They may also use practical examples of support based collaboration between municipal departments and partner organizations, which describe do’s and don’ts for reaching commitment and shared priority setting among public health parties. The guideline supports health promoters with evidence based interventions for (e.g.) obesity and depression, and offers formats for setting up regional programs for specific health risks, such as the prevention of alcohol abuse. Successful implementation of the guideline can be defined as ‘improved local collaboration in projects and programs for integrated health’ (e.g. environmental planning and stimulating physical exercise). In addition, the target population will be better reached by interventions based on evidence. The Guideline offers RHS organizations new methods that challenge their professionals to practice specific (partly new) competencies and skills.

This research aims to answer two questions:

1. To what extent do RHS professionals implement the guideline?
2. What determinants are associated with the implementation of this guideline?

### Exploring determinants of guideline implementation in local health policy

This study wants to gain leads for improving local public health policies that fit within the structures and workflow of local health organizations and their cross sectoral networks [8]. International research provides an extensive range of knowledge when it comes to barriers and facilitators for the implementation of clinical guidelines. Determinants have been found such as professionals’ views and beliefs [9], outcome expectancies of an innovation [10], knowledge and attitudes towards change of practice [11], self-efficacy beliefs [12], and social- and organizational support [13]. Less has been written about the determinants for guideline implementation within the political-administrative context of public health [14, 15].

Clinical guidelines often target rather homogeneous professional groups (e.g. doctors, paramedics) which is different from the implementation of guidelines for local health policy by a network of organizations. Besides, clinical guidelines predominantly prescribe a step-by-step treatment of a patients’ specific disorder with a specified outcome. The adoption process usually takes place in a hierarchical organizational context that often leaves little or no room for personal interpretation and flexibility. In contrast, the implementation of policy guidelines for local health needs to build coalitions among various organizations, each having their own interests, priorities and perceptions about the means by which public health goals are best achieved. Compared to a clinical setting, the process of adoption of policy guidelines in public health requires more negotiations among network partners for reaching consensus about shared goals and their investment for reaching these goals [16]. As such, cross-sectoral collaboration in public health policy making requires a more horizontal basis with input from the participants’ calculations of their own costs-benefit ratio [17, 18].

Overall, the implementation literature since Matland (1995) [19] has come to a consensus about the need for combining both top-down and bottom-up strategies in order to account for the local conditions for guideline implementation, such as available resources, specific interests of coalitions, partners, activities already implemented and the distribution of power [15].

### Theoretical framework

**Fig. 1:**
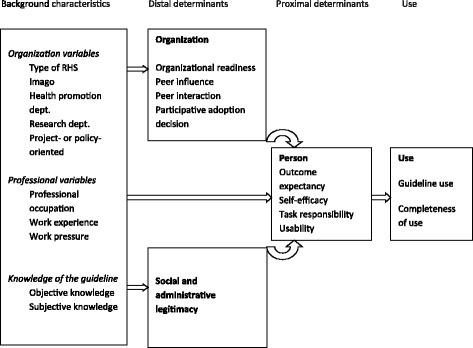
Research framework of determinants of guideline use

This research focuses on the guideline’s implementation by RHS policy advisors and health promoters. In order to assess implementation barriers and facilitators, we constructed a research framework of potentially relevant determinants of guideline use. The theory- and research-based framework MIDI (Measurement Instrument for Determinants of Innovations) was used as a point of reference for the framework for this study [11, 20]. MIDI offers an overview of potentially relevant determinants of public health innovations and leads for assessment. The framework for this study consisted of proximal determinants (e.g. task responsibility), which are expected to impact guideline use directly. The selected, distal determinants (e.g. years of working experience) are expected to be mediated by the proximal factors. The research framework was further refined by premises from Rogers’ diffusion of innovations theory [13], Bandura’s social cognitive theory (i.e. Self-efficacy theory) [12, 21], policy theory [22–24], organization theory [25, 26], and by the results of a recent Dutch study among key informants about local public health implementation processes (i.e. RHS professionals, RHS managers, public health experts, municipal policy officers, and guideline developers) [27]. The framework applied in this study is presented in Fig. 1.

## Methods

### Composition and check of internal consistency of determinants

Determinants, as described in the research framework, were assessed by one or more questionnaire items. They were assessed as either dichotomous, continuous, counts, or by Likert-type scaled items). Composite scores were computed when acceptable levels of internal consistency (Cronbach’s alpha ≥0.60) were reached. Composite scores were computed by dividing the sum score by the number of items. Negatively formulated items were flipped first, whenever necessary. Table 1 provides an overview of the Alpha scores.

**Table 1:** Number of items and internal consistency of the determinants

Determinants	Potential score	Number of items	Cronbach’s alpha
Person			
Outcome expectancy	1 (low) - 5 (high)	14	0.90
Self-efficacy (A)	1 (low) - 5 (high)	1	
Self-efficacy (B)	1 (low) - 5 (high)	1	
Task responsibility	1 (low) - 5 (high)	3	0.70
Usability of guideline	1 (low) - 5 (high)	19	0.89
Organization			
Encouragement of guideline use	1 (low) - 5 (high)	3	0.73
Organizational readiness	1 (low) - 5 (high)	6	0.61
Peer influence (peer support to use the guideline)	1 (low) - 5 (high)	4	0.68
Peer interaction (amount of meeting types in which guideline is addressed)	0–8	8	0.68
Participative adoption decision	yes vs no	2	1.0
Social and administrative legitimacy			
Legitimacy of the guideline	1 (low) - 5 (high)	9	0.63
Background variables			
Organization type	urban vs regional RHS	1	
Health promotion dept.	within RHS vs not within RHS	1	
Research dept.	within RHS vs not within RHS	1	
Imago of RHS	1–5 (positive imago)	2	0.74
RHS orientation	1 (project oriented) - 5 (policy oriented)	1	
Professional occupation	health promoter vs policy advisor	1	
Work experience	number of years	1	
Work pressure	1 (too low) - 5 (too high)	1	
* Knowledge of guideline*		7	0.65
Subjectively	0–2 (knowledge of availability and concept)	2	0.60
Objectively	0–5 (knowledge of content)	5	0.80

### Outcome measures: Use and completeness

‘Guideline use’ was defined as whether or not the respondent used any of the health topics and/or checklists. ‘Completeness of use’ was defined as the number of the health topics (range 1–5) and/or checklists (range 1–5) included in the guideline, that were put into practice. Because ‘guideline use’, and ‘completeness of use’ are quite distinct phenomena, it was expected that they could be affected by different factors.

### Proximal determinants

‘Outcome expectancy’ was assessed as the product of perceived importance and feasibility of the guidelines’ key objectives. We used 14 5-point Likert scaled items. ‘Self-efficacy’ was assessed by two five-point scaled items. ‘Task responsibility’ contained three items, such as ‘The guideline contains activities that fit my job within the RHS’. For all Likert scaled items, response options ranged from 1 = strongly agree to 5 = strongly disagree. We used 19 items to assess ‘usability’.

### Distal determinants

Measurement of ‘organizational readiness’ contained six 5-point scaled items. We used four 5-point scaled items for measuring social influences. ‘Peer interaction’ referred to the number (1–8) of different meetings in which the guideline’s implementation was discussed. (e.g. a section meeting of RHS policy officers, or a meeting between RHS manager and municipal city councilor). ‘Participative decision making’ about the guideline’s adoption was assessed dichotomously by two items, such as ‘Were executive professionals involved in the decision whether or not to use the guideline within the RHS?’).

Perceived ‘social and administrative legitimacy’ was measured by nine 5-point scaled items, such as ‘Our municipalities encourage to work in accordance with the recommendations of the guideline’.

### Background characteristics

‘Organizational background characteristics’ referred to RHS type (urban vs. regional). ‘RHS’s imago’ was assessed by two 5-point scaled items such as ‘My RHS has a positive image among the municipalities’. To assess the policy support orientation of the RHS, we used one 5-point scaled statement. ‘Professional occupation’ required one answer (tick box: executive health promoter, health policy officer, team leader, manager, and other). We used an open gray box for ‘working experience’ (in years), and five options for ‘perceived work pressure’ (1 = too low, 2 = low, 3 = moderate, 4 = high, 5 = too high). For measuring ‘subjective knowledge of the guideline’, we used three 5-point scaled questions (e.g. ‘I know where to find the guideline’). ‘Objective knowledge’ (of guideline content) was assessed by five 3-point scaled items, like ‘The guideline contains methods for social marketing to enhance political - and administrative base of support’ (1 = yes, 2 = no, 3 = don’t know).

(See Additional file 1: Appendix 1 with questionnaire variables and questions)

### Analysis

First we assessed the univariate associations between guideline use and the determinants in our framework by means of Chi-squared tests for nominal variables, non-parametric Mann-Whitney U Test for ordinal variables, and T-tests for continuous variables. Only determinants associated with guideline use (*p* < 0.1, two sided) were entered in the multivariate logistic analysis (forward selection). Finally, mean-score differences between users versus non-users of the guideline were computed for each individual item of the determinant(s) in the final multivariate model.

A similar stepwise multivariate linear regression approach was used for analyzing the determinants of completeness of use.

## Results

### Respondents’ characteristics

The questionnaire was sent to 304 regional public health professionals, and was returned by 120. For identification of potential respondents we used the national RHS department address files. These files contained addresses of RHS functions (such as managers and former employees) who were not meant to (and actually did not) respond to the questionnaire. A check with all RHSs revealed that RHS organizations had insufficient insight into the exact number of policy advisors. Respondents with functions other than policy advisor or health promoter, 14 in total, were excluded from analysis. Also excluded were 33 respondents who returned incomplete questionnaires (i.e. without information on primary outcomes). Our final dataset included 73 complete cases and all (28) Dutch RHS organizations were represented by at least one professional.

### Outcome: Guideline use and completeness of use

Of all respondents, 35 used the guideline, 38 did not. Among the 35 respondents who used the guideline, thirty-one respondents had used at least one health topic. The topic most often used was obesity (*n* = 19), followed by alcohol (*n* = 11), smoking (*n* = 6), sexual health (*n* = 6) and depression (*n* = 4). The checklists for policy planning were used by 14 respondents. Use of checklists was highest for checklist B (policy preparation; *n* = 10), followed by C (policy formulation; *n* = 9), E (policy preconditions; *n* = 7), D (policy execution and evaluation; *n* = 6) and A (policy context; *n* = 5). These results showed that, except for the topic ‘obesity’, guideline use by RHS professionals was rather moderate.

Of the user group, 18 used only one public health topic or one checklist and 17 used 2 to 7 topics or checklists. The average score for completeness of guideline use was 2.37 (sd = 1.78; range 1–7).

### Internal consistency of the determinants

Cronbach’s alpha ≥0.60 was used as cut off point for internal consistency of the composite variables. These internal consistency checks led to two adaptations: 1) the two self-efficacy items didn’t correlate well enough (alpha 0.46) and were therefore analyzed as separate factors (Self-efficacy A: ‘The guideline contains methods and tasks which I can actually perform’; Self-efficacy B: ‘I don’t think I can exchange my own routines with the new methods prescribed by the guideline), and 2) ‘Organizational readiness’, (9 items, alpha = 0.50) was split into two subscales, labeled as ‘Encouragement’ (referring to the presence of deliberate activities to promote guideline use) and ‘Organizational readiness’ (referring to the presence of interdisciplinary communication and sharing of knowledge and aspirations on integrated health targets in the RHS’s hierarchy). The alpha scores for the final constructs ranged from 0.61 to 1.0 (Table 1).

### Explaining guideline use

The univariate associations found between determinants and guideline use are presented in Table 2. Of the background characteristics, only subjective knowledge and objective knowledge appeared associated with guideline use (*p* < 0.10). Of the proximal and distal determinants, only perceived task responsibility and usability were significantly related to guideline use (*p* < 0.05). The inter-correlation between these two determinants appeared to be moderately high: *r* = 0.65 (*p* < 0.001).

**Table 2:** Determinant scores according to guideline use

Determinants	Outcome measures	Total (*n* = 73)	users (*n* = 35)	non-users (*n* = 38)	*p*
Person					
Outcome expectancy	mean (SD)	3.15 (0.65)	3.17 (0.58)	3.14 (0.71)	0.93
Self-efficacy (A)	mean (SD)	3.90 (0.85)	4.00 (0.77)	3.82 (0.93)	0.42
Self-efficacy (B)	mean (SD)	3.89 (0.91)	3.91 (0.78)	3.87 (1.02)	0.97
Task responsibility	mean (SD)	4.09 (0.81)	4.30 (0.71)	3.89 (0.85)	0.028*
Usability	mean (SD)	3.80 (0.50)	3.97 (0.36)	3.64 (0.55)	0.002*
Organization					
Encouragement	mean (SD)	2.12 (0.94)	1.99 (0.92)	2.25 (0.95)	0.15
Organizational readiness	mean (SD)	3.08 (0.66)	3.10 (0.60)	3.06 (0.71)	0.52
Peer influence	mean (SD)	3.06 (0.76)	3.15 (0.84)	2.97 (0.66)	0.29
Peer interaction	mean (SD)	1.84 (1.68)	2.09 (1.58	1.61 (1.75)	0.14
Participative adoption decision	%	42.5	48.6	36.8	0.31
Social and administrative legitimacy					
legitimacy (mean (SD))	mean (SD)	2.84 (0.45)	2.82 (0.53)	2.86 (0.36)	0.68
Background variables					
Organization type: urban RHS	%	5.5	2.9	7.9	0.67
RHS with research dept.	%	42.5	45.7	39.5	0.59
RHS with health promotion dept.	%	68.5	62.9	73.7	0.32
Imago	mean (SD)	3.68 (0.69)	3.67 (0.73)	3.70 (0.66)	0.75
Project/policy-oriented	mean (SD)	2.82 (1.09)	2.86 (1.19)	2.79 (0.99)	0.84
Professional occupation: health promoter	%	42.0	34.4	48.6	0.23
Work experience in years	mean (SD)	7.82 (6.78)	7.66 (6.29)	7.97 (7.29)	0.85
Work pressure	mean (SD)	3.60 (0.60)	3.60 (0.60)	3.61 (0.60)	0.96
Knowledge of guideline					
Subjectively (availability)	mean (SD)	1.92 (0.36)	2.00 (0.00)	1.84 (0.49)	0.06*
Objectively (content)	mean (SD)	1.49 (1.29)	1.77 (1.29)	1.24 (1.26)	0.09*

*p < 0.10

When objective knowledge, subjective knowledge, task responsibility, and usability were entered in the multivariate logistic model according to their theoretically expected order (forward selection), only ‘usability’ remained in the final model with OR 5.86 (1.68–20.5). The model fit (proportion of explained variance) appeared rather weak (Nagelkerke R Square 0.17).

For more in-depth insight into usability, as determinant of guideline use, we assessed the mean score differences of the 19 usability items between users versus non-users (Mann-Whitney *U*-tests). Table 3 only shows the mean scores differences that were statistically significant at *p* < 0.05.

**Table 3:** Mean score differences and standard deviations in perceived usability between users vs non-users of the guideline^a^

Behavioral beliefs (range 1–5)	Total (*n* = 73)	Users (*n* = 35)	Non-users (*n* = 38)	Significance of difference
	(mean (sd))	(mean (sd))	(mean (sd))	*p*
The guideline offers me a clear guidance for the development of local health (policy)	4.10 (0.89)	4.37 (0.77)	3.84 (0.92)	0.007
The guideline contains clear instructions for RHS application	3.67 (0.85)	3.91 (0.82)	3.45 (0.83)	0.010
I expect that collaboration with other sectoral policies actually leads to a more effective approach to the guidelines’ five health topics	4.41 (0.88)	4.66 (0.64)	4.18 (1.01)	0.025
I think the guidelines’ concepts are scientifically well-founded	3.86 (0.84)	4.17 (0.66)	3.58 (0.89)	0.002
I think the guideline offers a sufficient number of examples to work on my own	3.71 (0.86)	3.91 (0.82)	3.53 (0.86)	0.023
I think the stepwise approach of the policy cycle is quite useful in my RHS practice	4.00 (0.76)	4.26 (0.70)	3.76 (0.75)	0.005
The guideline provides sufficient flexibility for use in specific local contexts of RHS	4.07 (0.84)	4.31 (0.72)	3.84 (0.89)	0.012
I think RHS perspectives on developing local health are compatible with the guidelines’ perspectives	3.81 (0.76)	4.00 (0.64)	3.63 (0.82)	0.038
The guideline fits in well with current national policies, regulations and laws	4.14 (0.79)	4.34 (0.80)	3.95 (0.73)	0.018

^a^Items which showed no significant difference, referred to: ease of finding themes in the guideline, alignment with other policy instruments, fit with RHSs’ own policy instruments, acceptability of time required for preparing the application of the guideline, and the applicability of specific guideline components within their RHS organization

Beliefs showing relatively high scores among both users and non-users referred to effective collaboration with other sectoral policies on the health topics covered by the guideline (mean = 4.41), and perceived fit with current national policies, regulations and laws (mean = 4.14). We found relatively low scores on perceived procedural clarity of the guideline (mean = 3.67) and on the number of examples to work on your own with the guideline (mean = 3.71).

The largest differences between users and non-users were found in their perception of how well the guideline is based in science, and their perceived clarity of the leads offered by the guideline for developing local health policy.

### Explaining completeness of guideline use

Univariate analyses showed that completeness of use was only significantly associated with Self efficacy A (‘The guideline contains methods and tasks which I can actually perform’; Spearman’s rho = 0.36; *p* < 0.05) and Self efficacy B (‘I don’t think I can exchange my own routines with the new methods prescribed by the guideline’; Spearman’s rho = 0.44; *p* < 0.01). The inter-correlation of the two self-efficacy items was rather strong (Spearman’s rho = 0.54; *p* < 0.001). The multivariate linear regression on completeness of use (forward selection) showed that only Self efficacy B entered the model (β = 0.43, 95% CI: 0.24–1.70). The model fit (proportion of explained variance) was weak (R Square 0.18).

## Discussion

The main goal of this study was to explore the determinants of implementation of a public health policy guideline within Dutch RHSs, since these should be the primary target for strategies aiming to improve implementation.

The questionnaires of 73 respondents (24% out of 304 health professionals approached) appeared eligible for analysis.

About half of these respondents reported to use the guideline. The guideline was most often used within the context of the prevention of obesity. This corresponds to the relatively high priority of obesity prevention in both national and local public health policies in the Netherlands [28].

In the univariate analysis of guideline use, we found ‘knowledge’, ‘task responsibility’ and ‘usability’ (procedural clarity), and Self-efficacy to be related to the use versus non-use of the guideline. The analysis of determinants of guideline use also showed subjective and objective knowledge to be associated with guideline use (Table 1).

To improve implementation of the guideline, dissemination of knowledge about the guideline should be improved in municipalities and in regional health services. This was also confirmed by the interview results indicating that not all professionals and managers were aware about both the availability and the guideline’s core objectives. Besides media exposure, such as articles in professional journals, presentations online or at conferences, awareness can be increased by interpersonal communication. The latter provides the opportunity for exploring alternative plans for implementation that are tailored to the characteristics of the local setting in which the municipality and regional health service operate. The planning process should account for shared decision making by professionals and management within and between the local municipality and regional health service [29]. The implementation plan should clarify how application of the guideline fits with the current organization perspectives, vision and still existing methods and tools. Besides, the planning should account for feedback on progress, technical support, and training [30]. Internal communication and collegial interactions can be further initiated via online news channels and the organizations intranet.

The results showed differential perceptions among the professionals concerning their ‘task responsibility’ with regard to using the guideline. These differences reflect insufficient correspondence between the guideline-related tasks and objectives and their own perception of their professional task-obligations. If not, the outcome might as well express some sort of defensive response of those experiencing uncertainty about their competence relative to the execution of particular guideline- related tasks. Nevertheless, implementation of the guideline can be expected to improve by: 1) maximizing procedural clarity about the professionals’ core tasks and responsibilities within the context of the guideline [31]; 2) aiming at consensus among the professionals and managers within the RHS on tasks for which both disciplines are to be held responsible; 3) (individual) coaching and feedback on progress during the stage that the guideline is put into practice [32].

As yet, the results for the determinant ‘usability’ (including ‘procedural clarity’) indicate that the guideline does not provide the professionals with enough clarity about guideline-related tasks and responsibilities. Besides, the respondents differed in the extent to which they expressed their need for more explicit guidance and clarity, irrespective of their perceived importance of implementing the guideline.

This may reflect differences in perceived mastery of the professional skills involved when implementing the guideline as intended. This provisional conclusion is congruent with the overall low ‘self-efficacy’ scores we also found. Self-efficacy beliefs can be increased by ‘vicarious learning’: watching role models practicing the intended course of action [21]. This can be accomplished virtually, for example within a training session or by watching a video, and in practice, for example when junior professionals watch seniors performing the intended task. In addition, coaching can help to ensure that professionals gradually gain confidence in executing new tasks. This is also supported by literature on improving self-efficacy beliefs within the context of implementation of guidelines [33].

### Limitations

Our conclusions are only tentative, because they are based in cross-sectional data and a relatively low number of cases. The response was lower than expected and 47 respondents could not be included in the analyses. Non-response was partly due to the timing of the survey, which was conducted fairly short (nine months) after publication of the (renewed) guideline. For some Regional Health Services, there was no or at least limited opportunity to incorporate the guideline because of the 4-year life cycle of the planning of regional public health policy. So, some regions were in the mid-term of executing their previously planned strategy and were not yet ready for preparation of the proceeding strategy period. This would have been compensated, at least partially, if we had assessed intentional use in the near future.

Selection bias may have affected some of the outcomes if respondents who had used the guideline would have been more willing to complete the questionnaire. In that case the descriptive statistics (percentages and averages) could be biased. However, the main question of this research was to explore associations between variables which are known to be less vulnerable for selection bias [34].

The amount of explained variance may have been affected by the low number of items used for the assessment of particular constructs. Our intention to develop a questionnaire (based on our research framework) that was feasible to complete within a restricted timeframe, may have been at the expense of the stability of some assessments, especially those based in a single item. Also the scope of the criterion ‘completeness’ (of use) is not the optimum when thinking about guideline implementation as intended by the developers. Implementation is more than just the number of themes and/or checklists used in practice, for example it does not account for the number of relevant others (in or outside their own organization) also using the guideline neither for the quality of implementation.

## Conclusions

The results of our analyses indicate that knowledge, perceived task responsibility and beliefs about the guideline’s ‘usability’ are best discriminating professionals who use and not use the guideline. Hence, these are primary targets for improving the implementation of the Guideline for Local Health Policy. For improving completeness of guideline use, attention should be given to the RHS professionals’ self-efficacy.

## Additional file

Additional file 1: BMC HSR Appendix 1′. Appendix 1 Determinants, outcomes and corresponding questionnaire items. The file provides determinants and outcomes with the questionnaire items on which they are based. (DOCX 50 kb)

## Abbreviations

MIDI: Measurement Instrument for Determinants of Innovations
RHS: Regional Health Services
