# Supplementary material for: Implementation of a guideline for local health policy making by regional health services: exploring determinants of use by a web survey
Source: BMC Health Serv Res. 2017 Aug 15;17:562. doi: 10.1186/s12913-017-2499-2 (PMC5558707; doi:10.1186/s12913-017-2499-2)
Supplement: Additional file 1: — BMC HSR Appendix 1′. Appendix 1 Determinants, outcomes and corresponding questionnaire items. The file provides determinants and outcomes with the questionnaire items on which they are based. (DOCX 50 kb) [file 12913_2017_2499_MOESM1_ESM.docx]

| Appendix 1. Determinants, outcomes and corresponding questionnaire items.  'Implementation of a guideline for local health policy making by Regional Health Services: exploring determinants of use by a web survey'. | | |
| --- | --- | --- |
|  | **Determinants** | **Questionnaire items** |
| **Distal** | **Background variables** |  |
|  | ***Organization*** |  |
|  | Type of Regional Health Service (RHS) | Tick box: urban RHS or regional RHS |
|  | Health promotion dept. yes/no | *Which departments are present in your RHS?* |
|  | Research dept. yes/no | *Which departments are present in your RHS?* |
|  | Perceived imago | *Please indicate to what extent you agree with the following statements* |
|  |  | My RHS takes a positive imago in the municipalities  My RHS takes a positive imago among partners in Public Health |
|  | Project – or policy-oriented | My department's expertise in the project based approach to health programs is stronger than its strategic policy advisory for municipalities |
|  | ***Professional*** |  |
|  | Professional occupation | Tick box: health policy officer or health promoter |
|  | Work experience (years) | Tick box: number of years in this occupation |
|  | Work pressure | *How do you experience for yourself the work pressure within the Regional Health Service during the past six months?* |
|  | ***Knowledge of Guideline*** |  |
|  | Subjectively (availability) | *Please indicate to what extent you agree with the following statements* |
|  |  | 1. I know where to find the Guideline  2. I know for what purpose I can use the Guideline |
|  | Objectively (content) | *Which of these components, do you think, are part of the Guideline?* |
|  |  | 1 Stages of the policy cycle for the establishment of local health  2 Preconditions for organizing the local health policy  3 Methods for social marketing to promote administrative support base  4 Screening instruments to determine which policies can contribute in addressing a health problem  5 Proven effective intervention for preventing obesity |
| **proximal** | **Person** |  |
|  | Outcome expectancy (2 related questions, A, B, answered separately) | *A. Here are some key objectives of the Healthy Community Guideline. How important do you find these objectives in your work? If you don't use the guide, try to indicate the importance of these objectives for your organization*  *B. To what extent do you expect to achieve those objectives by using the Guideline? If you know the guideline only superficially, please base your expectations on your first impressions.* |
|  |  | **1**.The establishment of municipal health shows a clear systematic approach  **2**. Health Topics connect locally with current political issues  **3**. There is local consistency between interventions based on integrated policies  **4**. Cooperation of different sectoral policies of the municipality is established  **5**. Municipality, RHS and external parties provide structural contributions to integral health policy  **6**. Positively assessed interventions be used  **7**. Targets for municipal health policy are formulated SMART (specific, measurable, achievable, realistic, time scaled)  **8**. Political ownership in the issues to be addressed is created  **9**. Citizens and target groups are involved in municipal health policy  **10**. There is a clear division of tasks between RHS, municipalities and local organizations  **11**. Budgets of different policies that can contribute to integrated health are clustered  **12**. The manual has been integrated into municipal procedures  **13**. The manual has been integrated into RHS procedures  **14**. Regional collaboration on the manuals' health issues is strengthened |
|  | Self-efficacy | *Here are some statements about the content of the*  *Guideline. Please indicate to what extent you agree with the statement* |
|  |  | **A:** The guideline contains methods and tasks which I can actually perfor' (SE-A)  **B:** I don't think I can exchange my own routines with the new methods prescribed by the guideline (SE-B) |
|  | Task responsibility | *Here are some statements containing beliefs about the guidelines. Please indicate to what extent you agree with the statement* |
|  |  | **1** The Guideline contains activities that fit my job within the RHS  **2** I think my RHS should implement the Guideline for local health advisory, even if our municipalities are not ready to implement the Guideline  **3** I experience the use of the Guideline as a constraint of my professional liberty |
|  | Usability | *Here are some statements about the content of the*  *Guideline. Please indicate to what extent you agree with the statement* |
|  |  | **1** The Guideline offers me a clear guidance for the development of local health (policy)  **2** The Guideline contains clear instructions for RHS application  **3** The Guideline contains clear instructions for the positions and roles of the RHS in local health policy  **4** It's easy to quickly find information in the Guideline  **5** The Guideline provides good starting points for better alignment of local health on the national priorities of health policies  **6** I expect that collaboration with other sectoral policies actually leads to a more effective approach to the Guidelines' five health topics  **7** Next to my other duties, the use of the Guideline requires an amount of time in preparation and implementation that I consider unacceptably high  **8** The Guideline is well aligned with other regional health policy instruments  **9** I think the Guideline offers a sufficient number of examples to work autonomously  **10** My RHS organization can cope with the suggestions in the policy checklists of the Guidelines  **11** The Guidelines' integrated approach to health with the five cornerstones is well applicable within the RHS organization  **12** I think the Guidelines' stepwise approach of the policy cycle is quite useful in my RHS practice  **13** The Guideline provides sufficient flexibility for use in specific local contexts of RHS  **14** I would welcome if the Guideline took more account of my RHSs' own instruments for local health policy  **15** The Guidelines' recommended health interventions are very well applicable in the local circumstances of our municipalities  **16** I think RHS perspectives on developing local health are compatible with the Guidelines' perspectives  **17** The Guideline fits in well with current national policies, regulations and laws  **18** I expect that collaboration with other policy sectors actually leads to a more effective approach to the Guidelines' five priority health topics  **19** I think the Guidelines' concepts are scientifically well-founded |
|  | **Organization** |  |
|  |  | *Here are some statements about Guideline implementation in your RHS organization. Please indicate to what extent you agree with the statement* |
|  | Organizational readiness | **1.** Regarding ambitions and goals of integrated health, my department maintains a clear communication structure between managers, policy officers, and health promotion executives  **2.** My RHS facilitates entering into contacts between public health officials and municipal officials through meetings within and / or outside of office hours  **3.** To achieve integrated health, my RHS needs to adjust its methods for maintaining external relations with local authorities and public health partners  **4.** My department promotes cooperation between RHS -, and municipal management to work on cross-sectoral health policies  **5.** In my RHS, there is coordination between departments of Youth, Infectious Diseases, and Health Promotion on the opportunities for integrated policy development  **6.** Alignment between the departments of Youth, Infectious Diseases, and Health Promotion is a problem due to the physical distance between different locations |
|  | Peer influence | **1.** My colleagues find the Guideline not well applicable in practice  **2.** My close colleagues expect me to integrate the Guideline in my work.  **3.** My colleagues agree on how the Guideline should be used  **4.** Managers in my RHS fully endorse Guideline use by staff members |
|  | Peer interaction | *How often, since its release in November 2010, has the Guideline officially been discussed in the meeting structure of your organization?* |
|  |  | 1. Management meeting(s) 2. Teamleader meeting(s) 3. Policy officer meeting(s) 4. Health promotion officer meeting(s) 5. Health promotion executive meeting(s) 6. Meeting(s) with municipal policy officer 7. Meeting(s) with aldermen 8. Meeting(s) with Head of Municipal Dpt. |
|  | Participative adoption decision (yes/No) | 1. *Were executive professionals involved in the decision whether or not to use the guideline within the RHS?*  2. *Have the executive RHS professionals had a say in the internal uptake methods for the Guideline?* |
|  |  |  |
|  | Social and administrative legitimacy | *Here are some statements about environmental factors that may affect the use of the Guideline: Please indicate to what extent you agree with the statement* |
| **proximal** |  | **1.** Our municipalities encourage to work according to the recommendations of the Guideline  **2.** Our local policy officers find the prevention budget too limited for close attention to the Guideline  **3.** In our municipalit(y)(ies), insufficient budget for health promotion affects administrative support base negatively  **4.** When our municipal administrators have to weigh cuts, the disadvantage will affect Health Promotion rather than Youth health care  **5.** Local public health partners (such as Mental Health, Addictions, General Practices) have no objections to my RHS in the role of chain director  **6.** Our municipal policy officers make active use of the Guideline  **7.** Policy goals are aligned and evaluated at all organizational levels of communication between RHS, municipalit(y)(ies),and partners  **8.** The Health Inspectorate assessment on the use of Guideline interventions for local health encourages my RHS to implement the Guideline  **9.** There is an adequate alignment between national health institutes and my RHS on a clear division of roles in consulting municipalities |
|  | **Outcomes** |  |
|  | **Use** |  |
| **Dependent variables** |  | *Please indicate for each of the following Guideline policy checklists to what extent you (have) used the checklist as intended. Please tick one box on each line*  *Please indicate for each of the following health topics (Obesity, Alcohol, Depression, Sexual health) to what extent you (have) used the various component as intended in the Guideline?* |
|  | Guideline use yes/no | Respondent has used at least one theme or one checklist / or used neither |
|  | Completeness of use | Respondent has used a number of themes (max. 5) and / or checklists (max. 5); score 1-10. |
